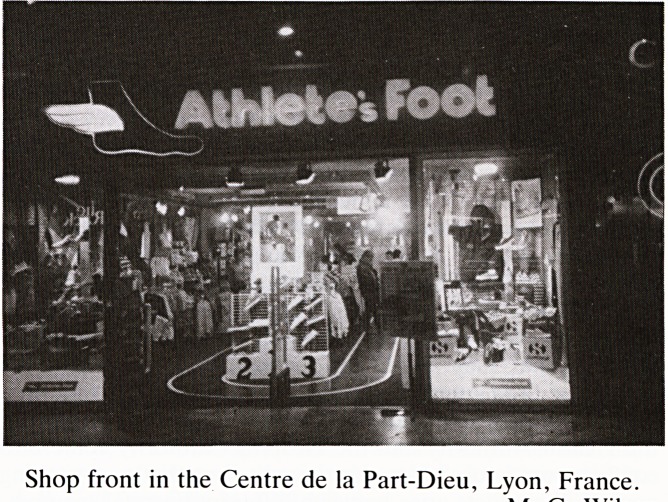# From Our Correspondents

**Published:** 1989-11

**Authors:** 


### From our Correspondents

### SOUTHMEAD AND THE NEW HEALTH SERVICE

Mr R. Hoyle, the District General Manager, gave a talk on October 26th to the staff at Southmead Hospital on the implications of the Government White Paper and the possible consequences as he saw them.

The essence of the new proposals is to separate the functions of the District Health Authority (DHA), as the purchaser of services on behalf of the community, from those of the Hospital as the provider of them. The DHA has to decide what services should be provided for the community and to purchase them wherever they can get the best bargain, and in this they have an obligation to take advice from the GPs. The Hospital authority is there to provide the best services it can and will be given a great deal of local autonomy in order to do so. Taking this concept to the full, a hospital can opt out of local authority control and manage its own affairs completely. It will be prepared to accept patients or provide services from any quarter and will receive payment according to a scale of negotiated charges.

The role of the Health Authority in implementing the proposals is, having decided what services the needs of the community demand, to obtain contracts from providers, not necessarily the local hospital. In doing this they will have to take into account other services such as the G.P. Services and the Social Services, which presumably cannot be bought elsewhere, and to consult with them. They will also have to consider their own direct management responsibilities, their financial probity and their performance and which of them e.g. catering and cleaning services etc. can be hived off.

He next dealt with some difficulties he foresaw in the application of this concept. Firstly, as there is only one purchaser and for most services only one provider there is virtually a monopoly situation and little real opportunity for competition. Secondly, the constraints of what is financially affordable are still ever-present. Thirdly realistic evaluations of the cost of patient services are very difficult to obtain, even more difficult in some cases is the real value and quality of these services and comparing them with those of other providers. He thought the way forward was probably that price negotiations would be local within a national framework.

He thought that for Southmead Hospital the need would be to develop services and encourage business from other Hospital authorities. there would be a difficulty from the DHA's point of view because sometimes they would be the interface between the purchaser and the provider. There will be real difficulties in drawing up satisfactory contracts between the purchasers and the providers. The DHA would have to decide how its management role would need to change and how much influence should be given to professional advisers.

Questions were plentiful. Could Mr Hoyle give examples of the actual services which he thought Southmead could provide competitively?—Showing a very proper caution he said he preferred not to reveal his management strategy, but when pressed he mentioned obstetrics, urology, renal dialysis and haematology. What about those aspects of the hospital's present services considered less saleable, would they be allowed to wither? Profits from the stronger services would not be able to develop the weaker ones because cross subsidy is not allowed under the rules. They would probably be used to develop other health care services. Was the answer to this question that they would be allowed to wither? Will the politics of the County Council affect decisions? He thought they probably would and that was very unpredictable. Could the Health Authority and the Hospital Authority come into conflict over issues? Yes, he thought, but that was nothing new. Will conflicts arise between different hospitals competing in the same District as providers of services? Again yes, but this need not be serious. What about teaching? There was the fear that hospitals may be able to cut costs by reducing staff whose role is largely a training one. Here he thought the Government would insist on the continuance of these posts.

Time ran out before the questions did. It was a very valuable session for those trying to think their way through the implications of this revolution. Most of us remember the last time the Conservatives attempted to reorganise the Health Service under Sir Keith Joseph, when a totally unnecessary Area Health Authority was created at enormous expense and resulting delay to other developments. That paper exercise was the result of advice from American management consultants and as we all know was later dismantled. Undeterred by that experience the government has again taken American advice in the planning of this 'experiment', reinforcing one's natural scepticism. However the uncompromisingly hostile attitude of the BMA and the unprecedented nature of its campaign against the proposals make it essential for the rest of us to try and think independently about it.

M. G. Wilson

### BRIAN JONES, Medical Librarian, Bristol University

Brian Jones retired after 19 years as Medical Librarian to Bristol University on 31st July. The Society owes him a great deal for his constant concern and willing involvement in our affairs. He not only gave meticulous care to our membership list and the regular dispatch of notices of meetings but so often fascinated and delighted us with exhibitions of the many rare and valuable antiquarian books which the Library is fortunate to possess and which few of us would see otherwise.

During this past 19 years there has been considerable development of the Library with increased space, a much larger stock of books and journals and the installation of more technological aids, self-service photocopying, medline, computerisation and most recently CD-ROM. With invariable courtesy our members have always been welcomed to the Library and Brian has been only too willing to demonstrate the mysteries of the latest gadget. His room has been continually cluttered with our unwanted journals and outdated medical books which have always been thankfully received.

His masterly handling of threatening situations at the Medical Library Sub-Committee when his budget has been cut could be a model for us all. Special thanks are due to him for his protection of our Society's interests with great tenacity

Brian Jones with one of his 'Treasures', – an original Vesalins 'De humani corporis fabrica' of 1542.

whether it be for increased Library opening hours, overnight loan of current journals, reserved car parking for our members or more finance for the Society's journal.

We shall miss Brian very much and wish him a long and happy retirement with time to pursue his special interests, perhaps at home with his computer trying to make his fortune on the Stock Exchange or enjoying the view gliding peacefullv above us.

Beryl D. Corner
Honorary Medical Librarian

### PHILOSOPHY AND MODERN MANAGEMENT

Nowadays everyone seems so concerned with everyone else's job. Is it measured or even measurable? How much ought it to cost? There is much philosophising and managing in the air. Alas, I am not a philosopher nor even a modern manager.

Some time ago one of the local University Committees sent around a circular advocating new times for academics. Briefly, the scenario ran as follows. Once a year everyone on the academic payroll should spend time defining their targets for the coming year. A questionnaire would be provided. Additional information can be added on separate A4 sheets at the end if need be. Discussions would follow with the Head of Department, and a year later a further talk should take place to see whether everything had been achieved. Fantastic.

### Rubbish

Well I ask you. Have you ever thought of what you are going to be doing in three weeks time, let alone a year hence? OK, some of you might say yes. For myself, I fully confess to being totally chaotic. Not that I feel in the least guilty. Different people work in different ways.

However just for a brief spell I tried being more organised. This involved amongst other things buying a decent sized wicker-work waste paper basket for the study, and dipping into Wittgenstein's *Tractatus logico-philosophicus* left in the house by an earnest Open University student. My, there is another world. I don't know whether you like thinking deep thoughts in the bath. I would, that is if they ever occurred to me. None the less all is there revealed in the *Tractatus*. For a start, had you ever thought '3.26 A name cannot be dissected any further by means of a definition: it is a primitive sign'?

No? Not yet. Join the club.

However my aims for the next academic year are:

1.001 To stop the cat sleeping in my new waste paper basket.
1.002 To stop the cat sleeping on my chair, when it's not in the waste paper basket.
. . .
1.037 To clear up the cat's hairs.
. . .
. . .
5.659 Not to waste everyone else's time with my personal administrative chaos.

There you are, five foolscap (sorry, A4) sheets to round off the form. Who would be a head of department? It must be a hard life.

Well, if this is the philosophy of modern administration, you can keep it to yourself. No wonder British universities are in a mess. Me, I'm going to have another warm bath with Wittgenstein.

PS. Did you know that '6.241 . . . the proof of the proposition $2 \times 2 = 4$ runs as follows:

$$(Q^{v})^{\mu x} = Q^{v x \mu} = \text{Def.'?}$$

Perhaps it's easier to be a manager. I promise not to worry about the cat.

Jack Davies

### 'BALL'S SIGN' OR 'BELL'S SIGN'

There has been discussion in the past concerning the association of rib fractures on a chest radiograph with alcoholism, particularly in the absence of a history of trauma – the so called 'Ball's Sign' (B.M.J. [1982] **285**, 1279).

I recently came across the case of a 76 year old gentleman who had a rather resistant bronchopneumonia who 'enjoyed an occasional drink'. There was no suspicion that he imbibed on a more regular basis until he kept his pyjama jacket on during the taking of one of his chest radiographs. This revealed a 'poacher's pocket' on the inside of the pyjama jacket which contained a miniature bottle of whisky! (see radiograph). Perhaps this interesting variant should be referred to as 'Bell's Sign'!

John Harvey

### JOGGING CAN DAMAGE YOUR HEALTH

Shop front in the Centre de la Part-Dieu, Lyon, France.
M. G. Wilson

## Figures and Tables

**Figure f1:**
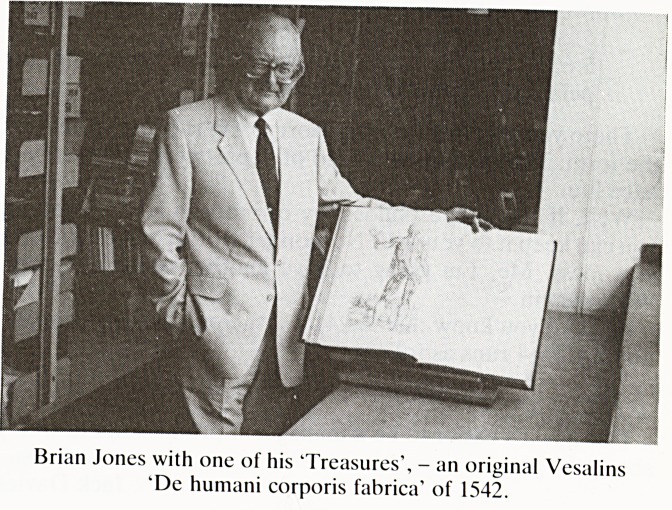


**Figure f2:**
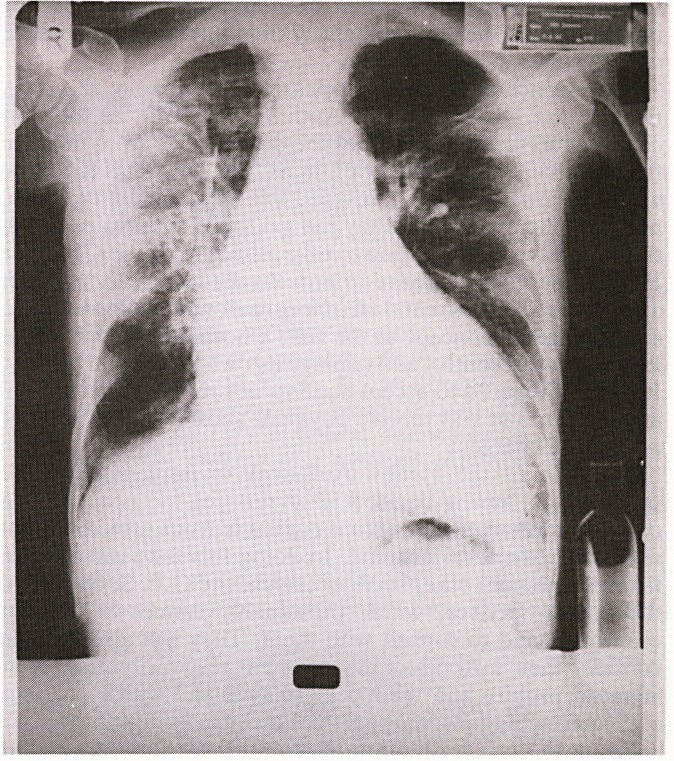


**Figure f3:**